# How environmental treaties contribute to global health governance

**DOI:** 10.1186/s12992-019-0493-7

**Published:** 2019-07-19

**Authors:** Jean-Frédéric Morin, Chantal Blouin

**Affiliations:** Université Laval, Québec city, Canada

**Keywords:** Environmental agreements, regime complex, sustainable development, global governance, global health governance, international institutions, treaties

## Abstract

**Background:**

Recent work in international relations theory argues that international regimes do not develop in isolation, as previously assumed, but evolve as open systems that interact with other regimes. The implications of this insight’s for sustainable development remains underexplored. Even thought environmental protection and health promotion are clearly interconnected at the impact level, it remains unclear how global environmental governance interacts with global health governance at the institutional level. In order to fill this gap, this article aims to assess how environmental treaties contribute to global health governance.

**Methods and results:**

To assess how environmental treaties contribute to global health governance, we conducted a content analysis of 2280 international environmental treaties. For each of these treaties, we measure the type and number of health-related provisions in these treaties. The result is the Health and Environment Interplay Database (HEIDI), which we make public with the publication of this article. This new database reveals that more than 300 environmental treaties have health-related provisions.

**Conclusions:**

We conclude that the global environmental regime contributes significantly to the institutionalization of the global health regime, considering that the health regime includes itself very few treaties focusing primarily on health. When reflecting on how global governance can improve population health, decision makers should not only consider the instruments available to them within the realm of global health institutions. They should broaden their perspectives to integrate the contribution of other global regimes, such as the global environmental regime.

## Background

Several scholars found that the architecture of global health governance is increasingly fragmented [1–4]. The number of stakeholders has risen sharply and includes non-governmental organizations (NGOs), private foundations, industrial groups and international research centers. Together, they contribute to the proliferation of various transnational actions, programs and partnerships. Far from centrally coordinating these flourishing activities, the World Health Organization (WHO) is one more actor, albeit an important one.

In the existing literature on global health governance, few studies analyze this fragmentation using the conceptual toolkit developed by scholars of international relations. In this article, we use the concept of “regime complex” to shed a new light on the transformations that have affected global health governance in recent decades. More specifically, we examine the health and environment regime complex and we argue that international environmental law makes a significant contribution to health governance.

The concept of “regime complex” arise out of the observation that there is not just one, but several international systems. The influential actors, the prevailing assumptions and the foundational institutions that govern the global trade system, for example, are quite different from those governing migration flows or the proliferation of weapons of mass destruction. To account for the specificities of each policy domain, international scholars use the concept of “international regime”, canonically defined as a “set of implicit or explicit principles, norms, rules and decision-making procedures around which actors’ expectation converge in a given area of international relations” [5].

Using this definition, global health appears as an international regime [6]. It includes international institutions devoted to infectious diseases surveillance, foreign assistance for health services, antibiotic resistance, and tobacco control, to name a few. Although these institutions have different members and functions, they share understandings of what health is about and of how health should be governed [7–9]. The global health regime includes, among others, the principle that policy decisions should be informed by evidence-based science, the norm that high-income countries should provide health-related assistance to low-income countries, the rule for states to notify the WHO in case of public health emergency of international concern, and the procedure of establishing transnational partnerships to address global health issues. As such, the global health regime has allowed a certain convergence of expectations among key actors [10].

However, international regimes do not develop in isolation. They evolve as open systems, which interact with other regimes in conflictual and synergic ways. There is no centralized world government and, therefore, no hierarchical ways available to arbitrate these interactions between regimes. To describe these complex situations, Kal Raustiala and David Victor coined the term “regime complexes”, which are defined as arrays of partially overlapping and non-hierarchical regimes [11]. This innovative concept invites anyone interested in understanding how a particular regime is created, how it evolves and how effective it is to consider other global regimes. For example, to understand global health governance, we should look at other areas of global governance, which may affect global health in positive or negative ways.

The dynamics that drive the creation of regime complexes are relatively well understood. In many cases, normative activities in one regime have unintended consequences for another regime [12]. For example, the investment regime restricted the ability of governments to adopt health regulations [13]. In some cases, regime interactions are deliberate. States can strategically move a specific problem from one regime to another, if the latter is perceived as being more receptive to certain interests or ideas. Developing countries adopted this strategy when they wanted to negotiate access to patented pharmaceutical products in the framework of the global health regime, rather than that of the intellectual property regime [14]. Likewise, international organizations seeking to expand their sphere of influence may choose to operate in a different regime to strengthen their position in terms of relevance, visibility or resources. This partly explains how the World Bank became involved in global health governance [15].

The consequences of regime complexes are generally less clear than their causes [16]. Overlaps between regimes can generate confusion, redundancy and inefficiency [17]. The existence of different competing institutions may strengthen already powerful actors in terms of their forum shopping strategy and exacerbate existing power imbalances [18]. On the other hand, institutional diversity and competition can favor a more flexible, adaptive and innovative form of governance [19, 20].

We know little about how regime complexity affects health governance. Some studies have convincingly argued that trade, investment, intellectual property and financial regimes have unintended negative effects on global health [21, 22]. However, the potential positive contribution of other regimes remains overlooked, with the possible exception of the human rights regime [23]. Some studies have shed light on the contribution that specific environmental treaties have made to health governance, particularly the Minamata Convention on Mercury and the Paris Agreement on climate change [24, 25]. However, no assessment has yet been conducted on the international environmental regime’s contribution to global health governance. By drawing on the concept of regime complex, this article aims to conduct an empirical assessment to determine the extent to which environmental treaties also contribute to global health governance.

## Methods

With the publication of this article, we are making the Health and Environment Interplay Database (HEIDI) publicly available. By making this new resource available, we hope the global health community will take it further and map the health regime complex.

The HEIDI dataset covers 2280 environmental treaties concluded between the eighteenth century and the present day (2017). All the treaties share three defining criteria: 1) they are binding under international law; 2) they were concluded by two or more states, 3) their primary purpose is to protect the natural world or develop the sustainable exploitation of natural resources. They include well-known multilateral treaties on biological diversity and climate change, but the majority are bilateral or regional treaties relating to issues, such as fisheries conservation, freshwater management, oil spill and nuclear waste. Most of the treaties were identified and collected by Ronald Mitchell (2003) [26].

We conducted a content analysis using the qualitative data analysis software NVivo to identify provisions linked to human health in these environmental treaties. We first used an extensive list of keywords relating to human health to identify a wide range of provisions. We reduced the initial sample, by applying a narrow definition of human health. For example, provisions on animal health, welfare, food supply and sanitary measures were excluded unless they explicitly referred to human health. Then, we classified genuinely health-related provisions into 14 different categories. We developed a detailed codebook with inclusion and exclusion rules for each of the 14 categories. We instructed a team of trained research assistants to read all 2280 treaties, using the codebook to identify any provisions that fitted our criteria. Different encoders analyzed the selected provisions to weed out any false positive results. Finally, to assess the frequency of false negatives, 10% of the treaties were coded a second time by a different encoder. Inter-encoder reliability for this double coding as measured by Cohen’s kappa is 0.706, which is considered a substantial level of agreement [27].

## Results

Using this method, we find hundreds of environmental treaties with health-related provisions. Not surprisingly, most treaties designed primarily to protect natural resources do not include provisions on human health. Environmental law and health law remain two distinct bodies of international law. However, no fewer than 338 environmental treaties include at least one health-related provision and some of them include up to seven of these provisions. Health-related provisions appears a total of 540 times in HEIDI.

### General trends

HEIDI reveals that health concerns entered the environmental regime much earlier than a focus on UN-sponsored activities might suggest. Although the 2013 Minamata Convention on Mercury and the 2015 Paris Agreement on climate change might be the most visible environmental treaties with health-related provisions, they build on a long heritage. For example, a 1903 treaty concluded among riparian states of the Rhine regulated the packaging, labeling and handling of substances that are dangerous for human health.

**Fig. 1:**
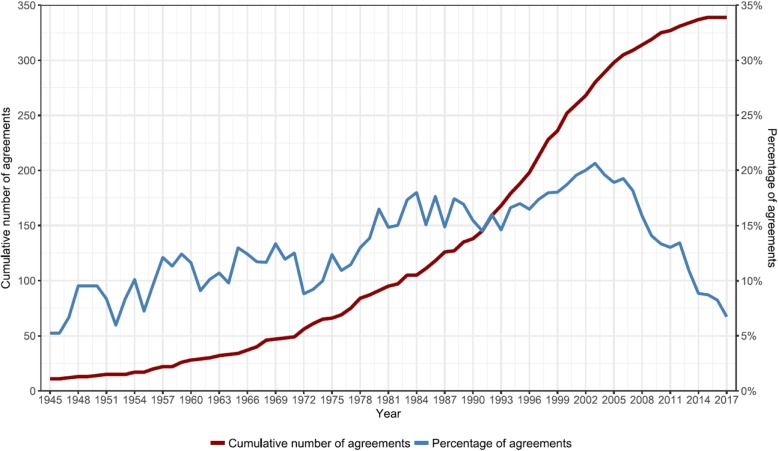
Growth in number of environmental treaties with health-related provisions

As illustrated by Fig. 1, the number of environmental treaties, including those with health-related provisions, rose rapidly in the 1970s. This period was characterized by growing ecological concerns, particularly in high-income countries. The ratio of new environmental treaties with health-related provisions over the total number of new environmental treaties peaked in the early 2000s. This was around the time of the 2002 Johannesburg Summit on Sustainable Development, where health was one of the central themes. Since then, the ratio is declining, but the cumulative number of environmental treaties with health-related provisions continues to increase.

We observe that the number of health provisions is strongly and positively correlated with the number of parties to an environmental treaty. In addition, multilateral treaties include more health provisions on average than bilateral treaties.

**Fig. 2:**
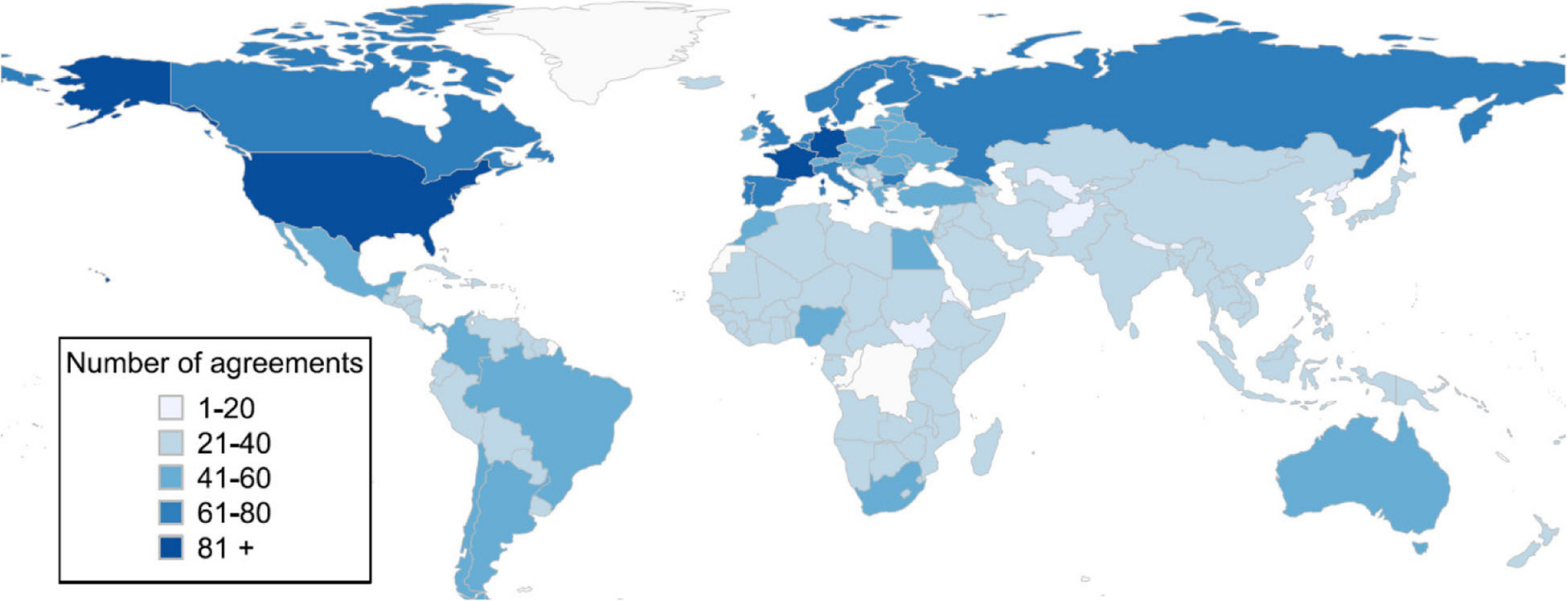
Number of environmental treaties with health-related provisions per country

However, as Fig. 2 shows, the geographical distribution of health provisions is irregular. On average, high-income countries have concluded more environmental treaties with health provisions than developing countries. Germany, France and the United States are part to more than 80 environmental treaties with health provisions. In contrast, almost all African and Asian countries have signed fewer than 40 environmental treaties with health provisions.

**Fig. 3:**
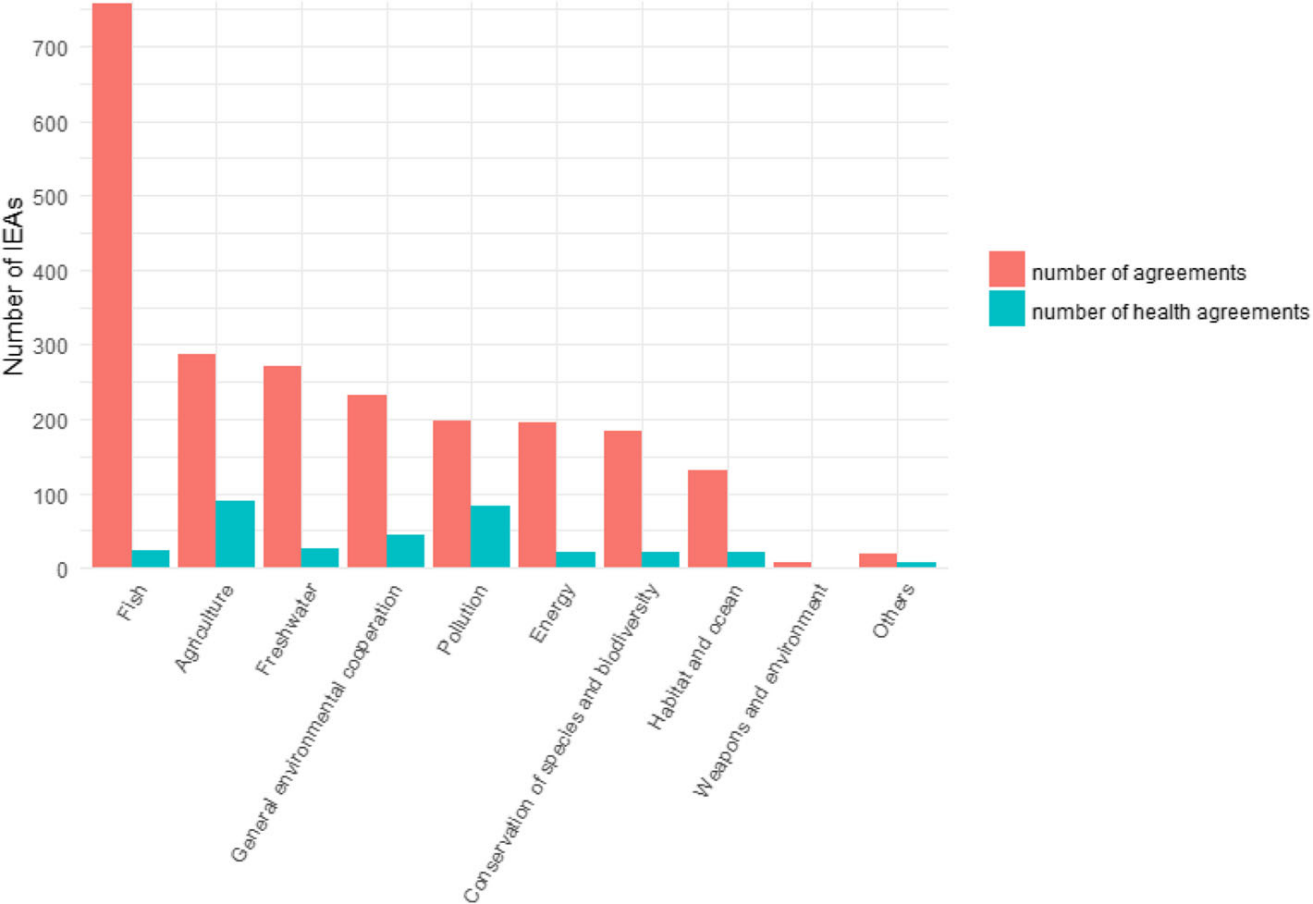
Number of environmental treaties per issue-area and number of treaties with health provisions per issue-area

In Fig. 3, we present the number of environmental treaties for the different issue areas and the number of treaties that include at least one health provision. We note that health provisions are more frequent in absolute terms in environmental treaties related to agriculture and pollution. Health provisions are unlikely to be found in environmental treaties devoted to fisheries or fresh water, even though these two issue-areas clearly have environmental health implications.

The fourteen categories of health provisions in HEIDI can be divided into three groups. First, we considered the provisions relating to the treaty’s general principles or objectives. The second group includes provisions about institutional matters, which particularly focus on how the treaty in question relates to global health institutions. The third group concerns the operational provisions, which are usually related to the implementation of the treaty. In this section, we describe the nature, frequency and distribution of these provisions.

### Principled provisions

We identified six categories of health-related provisions dealing with the general objectives. The most frequent type, with 150 occurrences, is a reference to human health in the environmental treaty’s preamble. For example, in the *1989 Basel Convention on the Control of Transboundary Movements of Hazardous Waste,* there is a reference to the awareness of “…the risk of damage to human health and the environment caused by hazardous wastes and other wastes and the transboundary movement thereof.”

In 97 treaties, we found causal statements about how a substance (e.g. radioactive material, inflammable substances or polluted water) or an activity (e.g. waste disposal) is considered dangerous to human health. For example, the *Convention on The Protection of The Marine Environment of The Baltic Sea Area* states “Pollution means introduction by man, directly or indirectly, of substances or energy into the marine environment, including estuaries resulting in such deleterious effects as hazard to human health…”.

In 96 treaties, we found provisions stating that protection or promotion of human health is one of the treaty’s objectives. The first time that human well-being or health was cited as an objective in an environmental treaty was in the 1972 *Convention for the Prevention of Marine Pollution by dumping from ships and aircraft*, an agreement between several European countries: “The Contracting Parties pledge themselves to take all possible steps to prevent the pollution of the sea by substances that are liable to create hazards to human health, […].”

A less frequent type of causal claims, found in 20 treaties, concerns statements about the importance of natural, biological and genetic resources for human health. The preamble to the 2010 *Nagoya Protocol on Genetic Resources* acknowledges “the importance of genetic resources to […] public health….”.

An important branch of international law concerns human rights and health services or living conditions that promote health. However, only 12 environmental treaties mention the right to health or the obligations of parties regarding the right to health. Most of the treaties that do so involve Russia or Eastern European countries, such as the 1997 environmental agreement between Belarus and Slovakia, which is also one of the first treaties to refer to “the human right to a healthy environment”. More recently, we also found references to the right to health in the preamble of the 2015 *Paris Agreement on climate change*. This provision was the result of concerted advocacy efforts from public health actors [28].

Another principled provision that refers to human health in environmental treaties is related to the precautionary principle. This is the duty to take action to prevent harm to the environment or human health, even when scientific evidence remains uncertain. There are more than one hundred references to the precautionary principles in the treaties in HEIDI, but only 13 of these provisions explicitly refer to human health. These provisions are usually found in treaties involving the European Union that were concluded in the 1990s and 2000s.

### Institutional provisions

Institutional provisions are less frequent than principled provisions. They are usually of a general order, as they do not specify exactly what they require from the parties involved or from the secretariat responsible for the treaties. Six environmental treaties require their parties to cooperate with the WHO and three include a requirement to cooperate with another health organization. For instance, the *1985 Vienna Convention for the Protection of the Ozone Layer* provides that “The Conference of the Parties shall [...] seek [...] the services of competent international bodies [...] in particular […] the World Health Organization.”

In other cases, the treaty may refer to the WHO (ten occurrences) or another health organization (three occurrences) without actually prescribing cooperation with them. This is illustrated by the *1999 Protocol on water and health to the Convention on protection of transboundary watercourses*, which includes a provision that mentions: “The quality of the drinking water supplied, taking into account the Guidelines for drinking-water quality of the World Health Organization”. We note that the probability of finding a provision on cooperation with WHO is higher in treaties involving jointly high-income and developing countries.

### Operational provisions

The third group of health-related clauses in environmental treaties relates to operational rules. Four types of clauses belong to this group. The most frequent type (61 treaties) are exceptions, which allow parties to the treaties to derogate from their treaty obligations for the purpose of protecting public or human health. For example, exceptions may include the right to kill an animal that endangers human lives or in times of famine, the right to access ports in the case of medical emergency (in fisheries agreements) and the right to impose more stringent measures to enhance human health protection. Such exceptions are particularly common in treaties that Canada is party to.

The second type of operational provisions, found in 54 environmental treaties, state that parties have the right to impose a quarantine for the cross-border trade of products. The majority of these provisions concern bilateral treaties between developing countries that were negotiated in the 1950s and 1960s.

The third type concerns clauses that commit parties to working toward harmonizing health policies. Ten environmental treaties call their parties to adopt similar guidelines, methods, policies, standards or procedures. The 1995 *Convention to Ban the Importation into The Forum Island Countries of Hazardous and Radioactive Wastes and To Control the Transboundary Movement and Management of Hazardous Wastes Within the South Pacific Region* specifies that: “The Conference of the Parties […] shall promote the harmoniation, at high levels of protection, of appropriate legislation, policies, strategies and measures for minimising harm to human health and the environment.”

Finally, we found four treaties in which parties commit to investing in health services and capacity building: the 1978 *Treaty for Amazonian Cooperation*; the 2001 *Stockholm Convention on persistent organic pollutants*; the *Framework cooperation agreement between Austria and Venezuela*; and the *2013 Minamata Convention on mercury*. This last treaty include the following commitments:

- (c) Promote appropriate health-care services for prevention, treatment and care for populations affected by the exposure to mercury or mercury compounds; and.
- (d) Establish and strengthen, as appropriate, the institutional and health professional capacities for the prevention, diagnosis, treatment and monitoring of health risks related to the exposure to mercury and mercury compounds.

## Discussion

We found that the environmental regime makes a significant contribution to the global health regime. In order to fully understand the global health regime, it is important to take into account other global regimes, such as the global environmental regime. The mapping presented in this article points to three key findings.

Firstly, we observe important links between the global health regime and the global environmental regime in terms of their principles and objectives. Negotiators of environmental treaties frequently include the protection of human health as one of their objectives. This type of linkage helps justify global collective actions on environmental issues.

Secondly, the linkages between the two regimes can also be analyzed with operational provisions. The majority of operational provisions found in environmental treaties aim to manage the potential conflicts that may arise between the two regimes. Various exceptions or safeguards allow states to arbitrate the regime interactions and prevent unintended negative consequences of the environmental regime on the global health regime.

Thirdly, we find few institutional provisions linking the two regimes. Environmental treaties seldom refer to the institutions involved in the global health regime and rarely build formal bridges with them. One explanation for this low level of institutional interaction may be due to the increasing number and diversity of institutions that are now involved in global health governance. Indeed, in such a fragmented regime, the different actors’ roles and responsibilities may not be very clear, especially for actors operating in other global regimes.

Some public health analysts might have expected stronger linkages between the global health regime and the global environmental regime, given the burden of diseases associated with certain environmental risks. The most striking case is air pollution, as it is associated with a high level of mortality and morbidity worldwide [29]. Yet, air pollution is not the subject of particularly intense rule-making in international environmental law. The trans-border nature of air pollution may not be sufficiently strong to create incentives to encourage international cooperation in this area. The sources of air pollution tend to be local and global interdependence is a powerful incentive for international cooperation, as shown by the large number of environmental treaties that focus on fisheries and transboundary water. In this sense, the fact that some of these treaties take into account health objectives comes as a surprise. While several observers of global governance express concerns for bureaucratic silos, policy incoherence, and negative externalities, the encompassing approach of some environmental treaties is an unexpected and welcome development.

## Conclusion

The current literature on the relations between the global health regime and other international regimes focuses on negative externalities. For example, studies have found that the trade regime, the foreign investment regime, the global finance regime, and the intellectual property regime accentuate health inequities [21, 22]. These negative side effects have led to calls for global governance processes that better protect policy space for health [30].

This article finds that other areas of global governance can make a positive contribution to global health. In particular, the global environmental regime includes more than 300 treaties with health-related provisions. This contribution is particularly significant considering that the health regime includes itself very few treaties. Moreover, other environmental treaties that do not explicitly refer to health might also contribute indirectly to health governance by reducing pollution levels and creating a healthier environment.

Taking into account various regimes’ positive contributions to global health is necessary to have a complete picture of the global health regime complex. Having such a complete picture is important because decision makers can potentially use instruments beyond the realm of global health institutions to improve population health. In addition, decision makers might want to transfer lessons from positive experiences to regimes that currently subordinate health under other policy objectives.

Future research on other regimes that affect global health governance should be extended. For example, an in-depth analysis of the health implications of the labor and the human rights regimes should be conducted, following the guidelines provided by the concept of regime complex.

## Data Availability

The Health and Environment Interplay Database (HEIDI) generated and analysed during the current study will be made available at  https://www.chaire-epi.ulaval.ca/en/data/heidi with the publication of this paper. Users of this dataset are asked to cite this paper as the main reference introducing the dataset.
